# Effect of sleep quality on repetitive transcranial magnetic stimulation outcomes in depression

**DOI:** 10.3389/fpsyt.2024.1458696

**Published:** 2024-09-23

**Authors:** Jamie Kweon, Andrew M. Fukuda, Asi P. Gobin, Lamaan Haq, Linda L. Carpenter, Joshua C. Brown

**Affiliations:** ^1^ Brain Stimulation Mechanisms Laboratory, Division of Depression and Anxiety Disorders, McLean Hospital, Belmont, MA, United States; ^2^ Department of Psychiatry, Harvard Medical School, Boston, MA, United States; ^3^ Neuromodulation Research Facility, Butler Hospital, Providence, RI, United States; ^4^ Department of Psychiatry and Human Behavior, Alpert Medical School of Brown University, Butler Hospital, Providence, RI, United States

**Keywords:** sleep, TMS, plasticity, predictors, depression

## Abstract

**Introduction:**

While repetitive transcranial magnetic stimulation (rTMS) is effective for 50-60% of those treatment-resistant depression, it is critical to identify predictors of response for optimal patient selection to improve therapy. Insomnia is a known symptom of depression that is both correlated with depression severity and associated with poor antidepressant response. Therefore, understanding this relationship may open new opportunities for the optimization of rTMS treatment. We aimed to explore whether baseline sleep quality, specifically insomnia, is associated with rTMS outcomes in a naturalistic sample of 975 patients (age 18-90; 63.9% F) receiving a standard course of rTMS treatment from two outpatient TMS clinics located within psychiatric hospitals in the United States. One site additionally collected information on concurrent medication use on 350 patients; among these, we examined whether pharmacological treatment of insomnia affected TMS treatment response.

**Methods:**

Depression was measured using the 30-item Inventory of Depressive Symptomology Self Report (IDS-SR) in site one and an abbreviated 16-item Quick Inventory of Depressive Symptomology (QIDS) derived from the IDS-SR in site two. Sleep disturbances were measured using three insomnia-related questions. Multilevel logistic regression was used to determine whether baseline insomnia scores were associated with TMS treatment outcome. Upon dichotomous categorization of the sample by insomnia and sleep-medication use, depression and sleep scores were analyzed across time using mixed repeated measures ANOVA.

**Results:**

We found that sleep quality improves after TMS (p<.001) and correlates with improvement in non-insomnia related depression symptoms (r= .318, p<.001). We found that among those who had significant insomnia at baseline, those not using sleep medications had significantly worse post-treatment IDS-SR scores compared to those using sleep medications (p=. 021) despite no difference in final insomnia score.

**Discussion:**

Together, our results suggest that while baseline insomnia is not associated with TMS effectiveness, treating insomnia may affect the trajectory of TMS therapy. Future prospective studies are needed to examine the effect of insomnia treatment alongside TMS for depression.

## Introduction

Though treatments for major depressive disorder have significantly improved over the years, we have yet to understand why certain individuals improve while others do not. For those who fail to improve with medications – estimated to be potentially up to 30% of patients (1), repetitive transcranial magnetic stimulation (rTMS) has emerged as a revolutionary therapy. However, even TMS response rates are limited to 45-60% with remission rates around 30% (2–4). Understanding who will potentially improve with TMS treatment may guide patient selection and provide insights into pharmacologic augmentation strategies (5).

rTMS is an FDA-cleared treatment for treatment resistant depression. During rTMS, a coil rests on the scalp and current is rapidly discharged through wire coils to generate a focused magnetic wave (6). Frequencies at 5-Hz or greater are believed to have excitatory modulatory effects (7), and are conventionally delivered to the dorsolateral prefrontal cortex to treat depression. At the neuronal level, 10-Hz rTMS appears to mediate effects through synaptic plasticity (8–12), with evidence in animals and humans that long-term potentiation (LTP) and LTP-like effects are produced (13).

Factors that influence plasticity, such as sleep, may therefore influence TMS outcomes. Literature has demonstrated changes in neural plasticity associated with sleep and depression (14). Sleep deprivation for example, has also been shown to disturb synaptic plasticity in both mice (15) and in humans (16). Sleep disturbances are a well-established symptom of depression that have been correlated with overall depression severity (17). Sleep quality has been previously studied as a promising predictor of treatment outcome across modalities, with research finding objective and subjective measures of sleep disturbance to be associated with poor depression treatment response (18–20). Therefore, understanding this relationship may open new opportunities for the optimization of rTMS treatment. Patients with depression have demonstrated abnormal REM sleep (21) and disrupted sleep architecture (22) associated with treatment outcome (23). Patients with comorbid insomnia and depression tend to require longer durations of treatment and lower remission rates across therapies (21), suggesting that poor sleep quality may affect the trajectory of treatment for depression.

Not only does insomnia potentially blunt improvement from depression therapies, but alleviating insomnia through targeted treatment has led to improvements in depression in patients with comorbid conditions. A sham-controlled trial found CBT-I with antidepressant treatments produced better outcomes than antidepressants alone (24). Furthermore, Watanabe et al. (2011) conducted a pilot study where patients with residual insomnia and depression despite antidepressants received CBT-I and saw significant improvement in both domains (25). A meta-analysis of 23 studies suggests a positive effect of insomnia treatment on depression outcomes, though interventions and populations were highly variable (26). Interestingly, sleep quality has also been found to facilitate plasticity processes such as changes in spine density (27). Considering 10-Hz rTMS has been demonstrated to increase spine size in mouse hippocampal slices (12), for example, this raises the possibility that better sleep could improve rTMS effectiveness through a shared mechanism. Taken together, it is plausible that sleep quality could modulate the trajectory of rTMS treatment for depression.

Despite the breadth of evidence that suggests insomnia influences depression treatment, current literature conveys mixed evidence on the influence of sleep quality on rTMS response. Lowe et al. (2013) found no relation between baseline insomnia or hypersomnia and rTMS treatment outcome in an analysis of data pooled from four clinical trials using rTMS treatment for depression (n=139) (28). Brakemeier et al. (2007) reported that patients (n=70) with worse baseline sleep had greater likelihood of TMS-related improvement; however, they were not able to replicate these findings in a follow-up study (29, 30). One study (n=195) initially found that early insomnia was related to worse outcome, but the result did not survive after adjusting for trial location heterogeneity (31). Together, these suggest baseline insomnia may not be a strong predictor of TMS treatment response. It is important to note, however, that Lowe et al. (2013) combined data from four trials all using varied frequencies, intensities, and number of sessions. These differing TMS parameters may enact disparate effects on brain networks (32), including those affecting sleep. Although other studies did control for location in analyses, data was pooled from 6 clinical trials that gave only 10 days of treatment (31), in comparison to the average 30-36 days in a standard rTMS treatment course. Finally, no study examined the potential role of sleep modulators, such as sleep mediations, and how this may have impacted rTMS outcome.

To address these gaps, we used naturalistic data with the largest sample size to date to parse out the role of insomnia in rTMS treatment response as well as investigating the role of sleep medications. We hypothesized that self-report baseline insomnia is not associated with clinical outcomes, per previous studies (28, 30, 31), but that pharmacologic treatment of insomnia improves rTMS effectiveness for depression.

## Methods

Data were retrospectively analyzed from the medical records of 353 naturalistically treated adult outpatients in the Butler Hospital TMS Clinic and 630 patients in the McLean Hospital TMS Clinic who received their first course of TMS with at least 30 sessions. Patient characteristics are reported in **Table 1**. Butler Hospital patients complete the Inventory of Depressive Symptomatology Self Report (IDS-SR) and Patient Health Questionnaire-9 (PHQ-9). McLean Hospital patients complete the Quick Inventory of Depressive Symptomology (QIDS) and PHQ-9. Both sites collected data before and after the course, and at interim time points throughout treatment (every 5 treatments at Butler, and every 10 treatments at McLean). All patients had a primary diagnosis of moderate-severe depression without psychotic features, inadequate or intolerable response to psychotherapy, and at least two (in most cases at least four) antidepressant and/or augmentation medications. Patients were evaluated by a psychiatrist specializing in mood disorders. Patients were on stable medication regimens before starting TMS and were instructed to keep regimens stable throughout the course of TMS.

**Table 1:** Demographic and clinical characteristics of three datasets.

Variable	Combined	Butler	McLean
n	980	350	630
Sex (% female)	63.9%	70.0%	60.79%
Age (in years)	48.5 ± 17.5	46.2 ± 15.6	49.8 ± 18.4
Responders (%)	40.9%	44.5%	38.5%
Remitters (%)	21.7%	25.5%	19.6%
10-Hz (%)	24.3%	68.0%	NA
5-Hz (%)	10.2%	28.6%	NA
18-Hz (%)	63.1%	NA	98.1%
Other dominant frequency (%)	2.4%	3.4%	1.9%
Pre-Tx QIDS Score	20.8 ± 6.18	22.8 ± 5.60	19.7 ± 6.21
Post-Tx QIDS Score	12.2 ± 7.24	12.9 ± 7.62	11.9 ± 7.00
Percent Change QIDS	-40.7 ± 32.9	-42.0 ± 30.9	-38.4 ± 34.0
Pre-Tx Insomnia Score	4.33 ± 2.10	4.60 ± 2.25	3.86 ± 2.19
Post-Tx Insomnia Score	2.88 ± 2.00	2.83 ± 2.27	2.78 ± 2.01

NA, not applicable.

### TMS protocol

On the first day of treatment, patients underwent a motor threshold procedure to determine the left hemisphere motor ‘hotspot’ and minimum stimulator intensity required to produce a finger twitch for >50% trials. At Butler, patients then began a standard 10-Hz treatment protocol delivering 3,000 pulses a day for 6 weeks, 5 times a week, followed by 6 sessions over 3 weeks. Butler patients were treated with a NeuroStar iron core coil (Neuronetics, 2003) over the left dorsolateral prefrontal cortex (dlPFC) at a stimulation intensity 120% of their resting motor threshold (rMT). In ~60% of cases (n=101), where patients had difficulty tolerating the 10-Hz protocol at 120% MT, they received 5-Hz stimulation (3,000 pulses). A minority of patients who transitioned to 1-Hz stimulation over the right dlPFC (12 patients received 1-Hz for more than 50% of treatment sessions) were excluded from our analysis. For a small number of patients without improvement, the total number of pulses per session was increased to 4000.

McLean TMS patients were treated on either a MagVenture B70 figure-8 coil (5% of patients) or a BrainsWay H1 coil (95%). The BrainsWay protocol entailed 18-Hz stimulation at 120% of rMT for 1980 pulses five days per week for 36 consecutive treatments. A small minority (~2%) of patients were switched from BrainsWay for tolerability. MagVenture protocols included intermittent theta-burst stimulation (iTBS) on the left, 1-Hz or continuous (c)TBS on the right, or bilateral. All Butler patients were outpatient, while 116 of the 630 at McLean began as inpatients.

### Clinical assessment

Clinical response was defined as a decrease in score by ≥50% from baseline to post-treatment. Remission was defined by a post treatment score ≤14 on the IDS-SR and ≤5 on the QIDS. As all QIDS items are included within the IDS-SR, a comparative QIDS score was also calculated for the Butler dataset. The three insomnia-related questions were determined as items 1 (Falling asleep), 2 (Waking up during the night and difficulty falling back to sleep), and 3 (Waking up too early). Each question had a score range from 0-3, with 0 representing no sleep disturbance (ex. “I never take longer than 30 minutes to fall asleep”) and 3 representing the most severe (ex. “I take more than 60 minutes to fall asleep, more than half the time”). The scores of these three questions were summated to create an “insomnia score” with a range from 0-9. Item 4 (Sleeping too much) was not included to separate insomnia from hypersomnia-like sleep disturbances. We then calculated an IDS-SR_25_ total score excluding insomnia items for analyses comparing insomnia to other depressive symptoms rated on the same scale, and likewise for QIDS to create a QIDS_13_. Previous studies (28, 29, 31) have used item-level sleep questions from the Hamilton depression rating scale (early (‘complains of nightly difficulty falling asleep’), middle (‘waking during the night except for the purpose of voiding’), and late insomnia (‘unable to fall asleep again if he/she gets out of bed’). Insomnia was defined as a score of 2 on at least two questions. Similarly, we used the 3 QIDS sleep questions to evaluate early, middle, and late sleep disturbances. Our methods are in line with those used in previous work, though we used a different depression scale. Of note, the QIDS-SR16 has demonstrated high correlation with the Hamilton scale (33).

### Sleep medications

Furthermore, we examined whether using sleep medications was associated with improvement in 1) sleep and 2) depression. Patients at both sites were instructed to keep medications stable through TMS. We included the following medications commonly prescribed for sleep impairment in our definition of sleep-aids: doxepin (Sinequan, Silenor), ramelteon (Rozerem), temazepam (Restoril), triazolam (Halcion), zaleplon (Sonata), zolpidem (Ambien, Zolpimist, Edluar, Intermezzo), eszopiclone (Lunesta), suvorexant (Belsomra). Three antidepressants- trazadone (Desyrel), amitriptyline (Elavil), and mirtazapine (Remeron) were also included, along with melatonin. Anti-anxiety medications were not included in our filter as we aimed to focus in on medications used more exclusively for insomnia. For Butler patients, we filtered through medication lists recorded on the first day of TMS treatment and those taking sleep medications during TMS were coded “1” and otherwise coded “0.”

To assess whether patients with insomnia taking sleep medications had clinical responses to TMS comparable to those without, we categorized patients into four groups by sleep quality x medication use: 1) No/low insomnia and not using sleep-aids (“-Insomnia -Meds”) 2) no/low insomnia using sleep aids (“-Insomnia +Meds”) 3) high insomnia and not using sleep-aids (“+Insomnia -Meds”) 4) high insomnia despite use of sleep-aids (+-Insomnia +Meds”).

### Statistical analysis

Categorical (responders, remitters) and continuous (percent and raw change in baseline to endpoint for IDS-SR, QIDS, and insomnia score) outcomes were explored with descriptive statistics. IDS-SR, QIDS, and insomnia scores were not normally distributed. Wilcoxon Signed Rank tests were used to determine within subject differences (baseline to post comparisons) and Mann-Whitney U tests were used to determine differences between groups in sleep quality and medication. Statistical significance was defined at p<.05 and two-tailed. We also analyzed correlations in sleep score with change in overall symptom improvement using Spearman correlation tests.

To determine whether baseline sleep scores predicted TMS outcome, we performed a multilevel logistic regression using the lme4 package (v1.1.33 (34);) with response as categorical outcome variable and baseline sleep, age, sex, and inpatient/outpatient status as fixed effects. Hospital site was included as a random effect to account for a potential influence of variance in the structured data. The above analysis was repeated with remission as outcome variable.

To create a categorial variable “sleep quality,” we chose the median score 4 as the cutoff score based on the histogram and descriptive statistics of baseline insomnia score (**Supplementary Figure 1**). Patients with baseline insomnia score with less than or equal to 4 were coded as 0 and those with a score greater than 4 were coded as 1. We then compared IDS-SR_25_/QIDS_18_ scores between no/low insomnia group and high insomnia group.

Kruskal Wallis Test was used to determine differences in baseline and post treatment IDS-SR and insomnia scores between the four groups. To analyze scores across time, mixed repeated measures ANOVA was applied. All statistical analysis was done in R (v4.3.1; R Core Team 2021).

## Results

### Demographics

**Figure 1:**
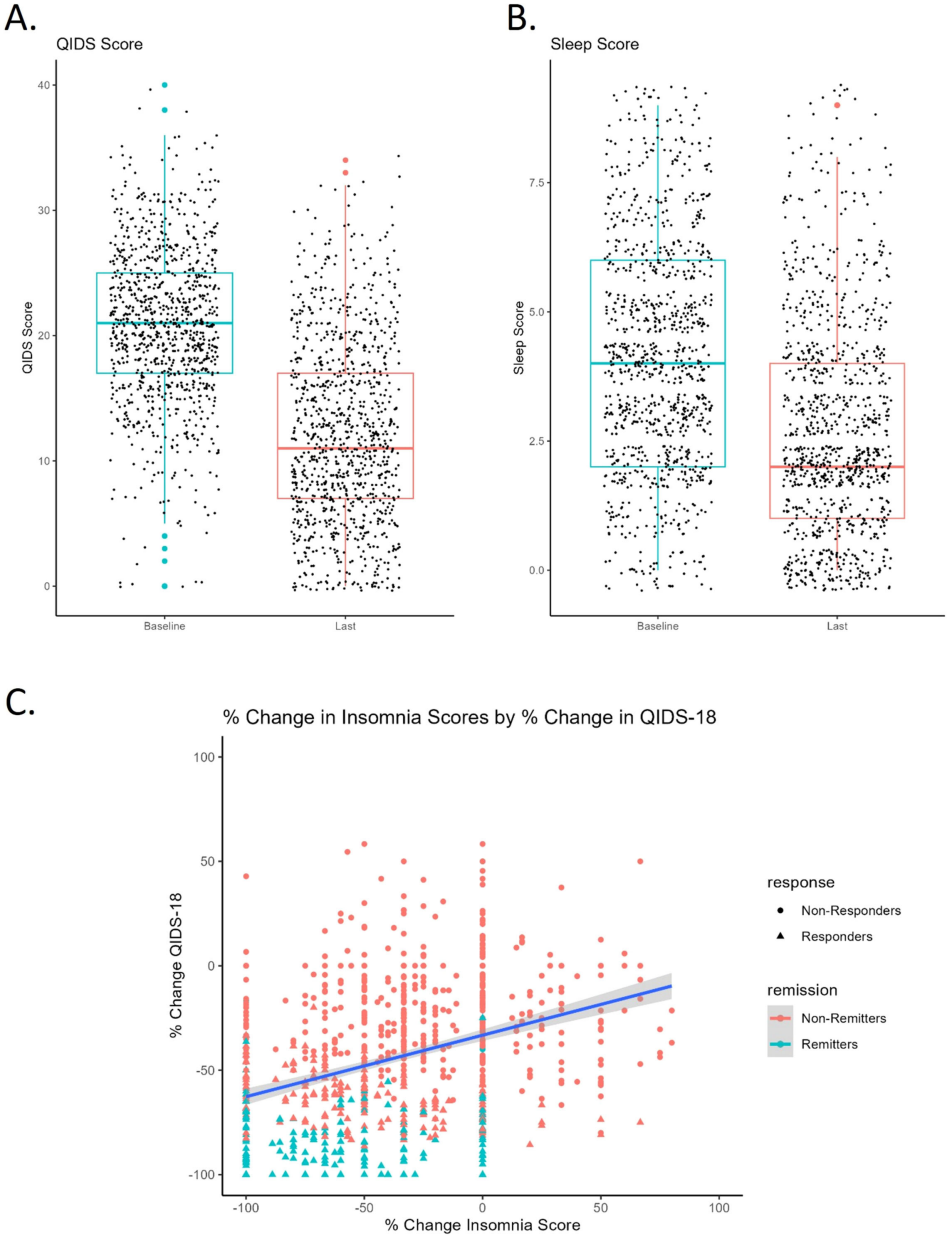
**(A)** Pre to post-treatment QIDS score with standard deviation (error bars) and individual data points. Colored dots represent outliers. Pre (20.8 ± 6.18), Post (12.2 ± 7.24). **(B)** Pre to post-treatment insomnia score with standard deviation (error bars) and individual data points. Colored dots represent outliers. Pre (4.33 ± 2.10), Post (2.88 ± 2.00). **(C)** Change in insomnia score positively correlates with change in QIDS_18_ score pre to post TMS. Negative percent change reflects improvement in symptoms (score reduction).

Between August 2016 and July 2022, 350 patients (245 F, age 18-84) completed a baseline and post rTMS questionnaire at Butler Hospital, and 630 patients (383 F, age 18-90) between October 2017 and April 2023 at McLean Hospital. Demographic and clinical characteristics are presented in **Table 1**. There was a significant decrease in QIDS score from baseline to post-treatment scores (Z= -24.3, p<.001, r= -.778, **Figure 1A**).

### Effect of TMS on sleep

Average baseline insomnia score was 4.33 ± 2.10. We found no significant differences by sex (Z= -1.24, p= .214, r= -.039) or correlation with age (r= .053, p= .094). Patients with greater baseline insomnia tended to have greater baseline QIDS scores, i.e., more severe depression (r=.57, *p*<.001). Insomnia scores significantly improved over the course of rTMS treatment (Z= -16.45, *p<*.001, r= -.519, **Figure 1B**).

After responses to the three insomnia-related sleep questions were subtracted, we found percent change in insomnia score correlated in a positive direction with percent change in QIDS_18_ (r=.318, *p*<.001, **Figure 1C**), indicating that sleep improved alongside general improvement in depression after TMS.

### Baseline sleep and TMS clinical outcome

To determine whether baseline sleep disturbance was associated with TMS response or remission rates, we first examined whether baseline and final insomnia/QIDS scores differed between responders and non-responders. Responders had a significantly higher initial insomnia score than non-responders (Z= -2.54, *p*=.011, r= -.081). After TMS, responders had significantly lower insomnia scores than non-responders (Z= -12.64, *p*<.001, r= -.404), as well as greater decrease in insomnia score as measured by percent change (Z= -14.42, p<.001, r= -.467). By contrast, remitters had significantly lower insomnia scores at baseline (Z= -3.11, p=.002, r= -.098) which persisted after TMS (Z= -13.84, p<.001, r= -.443).

A binary logistic multilevel model (MLM) with responder status as dependent variable and baseline insomnia, baseline QIDS18 score, sex, age, and inpatient status as fixed effects, controlled for location, did not show a significant effect of baseline insomnia on response status. Similarly, a logistic regression model using baseline sleep to predict remission status with the same covariates showed no significant effect of insomnia on remission outcome when QIDS_18_ was included. Baseline insomnia did not predict TMS treatment outcome.

### Insomnia as a modulator of TMS outcome

**Figure 2:**
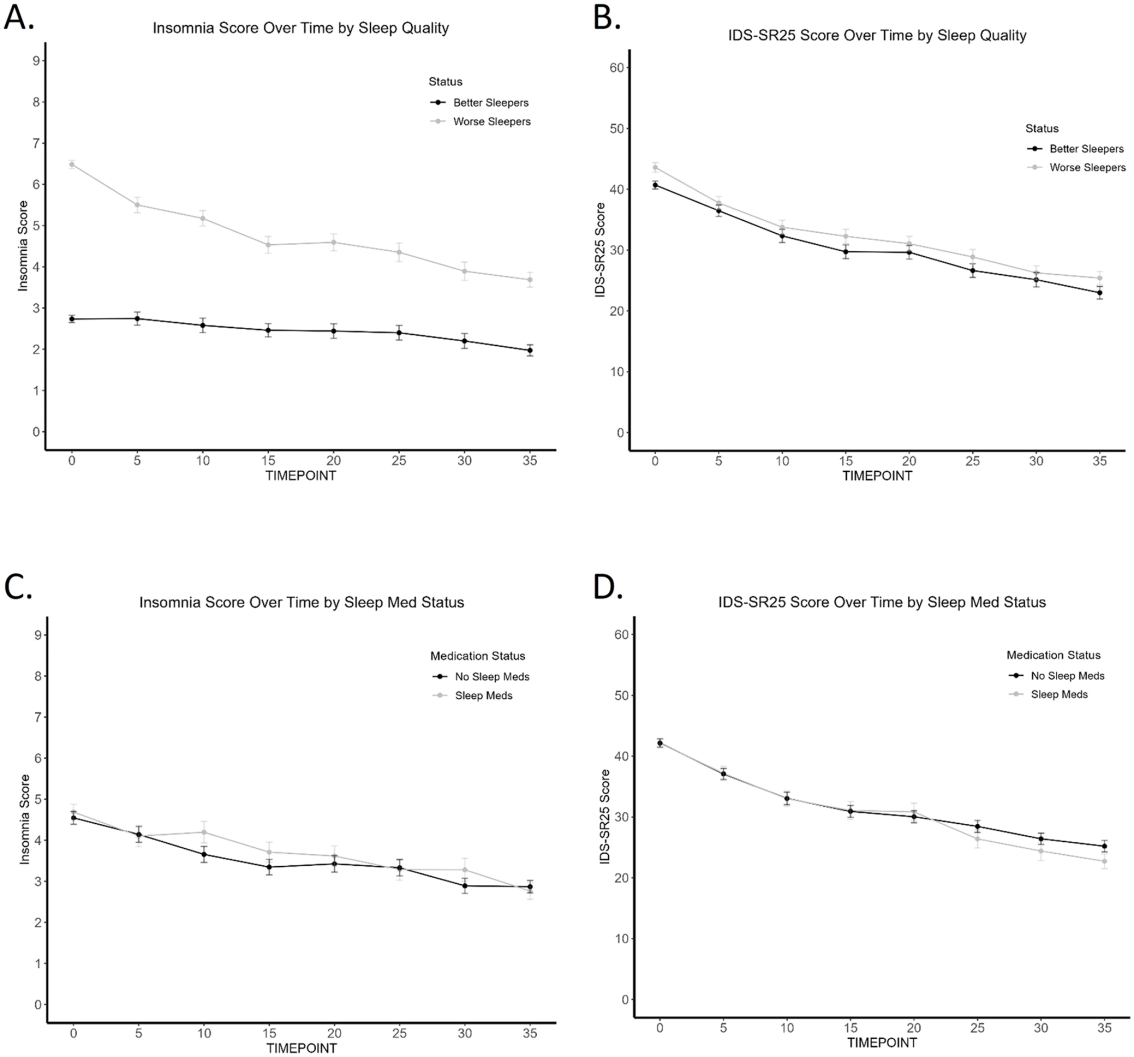
Trajectory of Insomnia and IDS-SR_25_ score over TMS treatment course (Butler). **(A)** Worse sleepers have consistently worse sleep scores across TMS treatment course. **(B)** Worse sleepers have consistently worse IDS-SR_25_ scores across TMS treatment course. **(C)** No difference in insomnia scores across TMS treatment course by sleep medication use. **(D)** No difference in IDS-SR_25_ scores across TMS treatment course by sleep medication use.

We went on to explore whether baseline insomnia influences the trajectory of symptom improvement. Using the Butler data set, insomnia and IDS-SR_25_ scores were plotted every 5 TMS sessions by better and worse sleepers. We found that patients with initially no/minimal insomnia have consistently lower scores for both insomnia and IDS-SR_25_ score across treatment course (**Figures 2A, B**). This difference was found to be significant by mixed repeated measures ANOVA, which produced significant effects of time (F(3.46, 443)=142.2, p<.001, η^2^= .03), quality (F(1,128)=5.176, p=.025, η^2^= .23), but not time by quality interaction.

### Sleep medications

To account for the use of sleep medications and their impact on sleep in our naturalistic sample, we found that patients taking sleep medications did not have significantly different baseline/final insomnia or IDS-SR_25_ scores when compared to patients not taking sleep medications. 243 (69.4%) participants in the Butler cohort were taking antidepressants at the start of TMS treatment. 144 (41.1%) patients were taking one or more of the sleep medications included in our analysis. **Figures 2C, D** show no separation in sleep scores and overall depression scores, respectively, between the two medication groups. Regardless of whether sleep medications were used, TMS treatment improved insomnia (effect of time; F(4.76, 609.44)=20.02, p<.001, η^2^=.04) and other depression symptoms (effect of time; F(3.53, 451.46)=140.82, p<.001, η^2^= .22). Sleep medication status was not associated with response or remission outcome, based on chi-square analysis.

### Insomnia and medication use

Finally, we sought to determine whether sleep medications influence treatment outcome through modulation of sleep. **Table 2** shows the number of patients in each group, mean sleep score, and mean IDS-SR_25_ score before and after TMS. Mixed ANOVA reveals that within patients with no/minimal insomnia, there is no difference in insomnia scores across TMS course between patients taking or not taking sleep medications (F(1, 76)= .208, *p*=.65, η^2^= .002, or in patients with insomnia by medication status (F(1,72)= .115, *p*=.736, η^2^=.001).

**Table 2:** Breakdown of the four sleep quality by sleep medication use groups.

Status	N	BL Insomnia Score	Last Insomnia Score	BL IDS-SR25 Score	Last IDS-SR25 Score
-Insomnia -Meds	144	2.82 ± .103	2.10 ± .165	41.0 ± .806	23.2 ± 1.25
-Insomnia +Meds	63	2.59 ± .159	1.73 ± .231	40.1 ± 1.10	22.6 ± 1.83
+Insomnia -Meds	95	6.62 ± .138	3.80 ± .236	43.6 ± 1.11	27.6 ± 1.43
+Insomnia +Meds	81	6.32 ± .145	3.56 ± .275	43.6 ± 1.08	22.8 ± 1.63

Number per group, and mean scores ± standard error of the mean for each measure. BL, Baseline; IDS-SR25, Inventory of Depressive Symptomology (Self-Report) without sleep items; “-Insomnia -Meds”, No/minimal insomnia without hypnotics; “-Insomnia +Meds”, No/minimal insomnia with hypnotics; “+Insomnia -Meds”, Insomnia without hypnotics; “+Insomnia -Meds”, Insomnia with hypnotics”.

**Figure 3:**
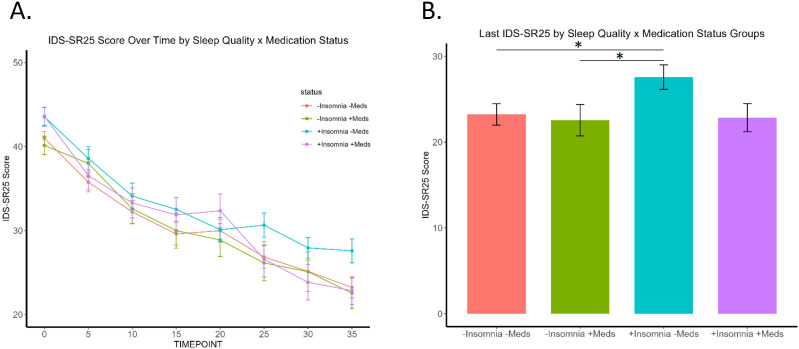
Trajectory of patients by the four sleep quality and medication use groups. **(A)** Significant effect of group (*p*=.04) and time (*p*=.01), but not group x time interaction. **(B)** “+Insomnia -Meds” group shows significantly higher final IDS-SR25 score at timepoint 35 compared to "-Insomnia -Meds" (p= .032) and "-Insomnia +Meds" (p= .039) groups. * signifies p <.05.

Examining IDS-SR_25_ scores over time by sleep quality group (no/low vs. high insomnia) and hypnotic medication use status (**Figure 3A**) revealed that patients taking sleep medications demonstrated no significant difference to those with healthy sleep and not taking sleep-aids. Notably, while the high insomnia group taking sleep medications had significantly higher baseline IDS-SR scores than patients with no/low initial insomnia, all three groups ended at comparable final depression scores. This effect was not due to insomnia improvement, as they had higher insomnia scores at the end of treatment. In contrast, patients with high insomnia not taking medications showed the least overall improvement with significantly higher IDS-SR_25_ scores post-TMS than the other three groups (**Figure 3B**). We found an overall significant effect of group (F(3,4)=7.37, p= .042, η^2^=.79, time (F(7,28)=3.28, p=.011, η^2^=.21, but not group x time interaction, with significant Kruskal-Wallis at final time point 35 (*p*=.04). Patients using sleep medications appear to have greater improvement in overall depression symptoms, even if insomnia does not significantly improve.

## Discussion

We found that patients using sleep medications see improvement in overall depression even if subjective sleep measures do not significantly improve. Upon categorization by insomnia severity and sleep medication use, patients using sleep medications show a drop in IDS-SR score and end at a similar position to better sleepers. In line with our hypothesis, treating insomnia appears to enhance TMS effectiveness, while baseline sleep quality does not predict TMS treatment outcomes, in line with the majority of other studies that have examined sleep as a predictor of response and found no relation between the two measures (28, 30, 31).

One potential underlying link between sleep, depression, and TMS treatment is synaptic plasticity. Impairments in the brain’s ability to reorganize and respond to changing stimuli, such as through LTD in the prefrontal cortex, have been implicated with depression (35). Studies in both animals (12, 36) and humans (8, 37, 38) suggest that rTMS enacts therapeutic change through the restoration of impaired synaptic plasticity in patients with depression. Interestingly, sleep has been found to modulate synaptic plasticity directly. For example, in a study using high-frequency electrical stimulation in the motor cortex of rats to induce synaptic plasticity, effects were partially occluded after prolonged wakefulness, and then restored after sleep (15). Similar results were found in humans; one night of sleep deprivation blunted the facilitatory effect of paired-associative stimulation (PAS) on motor-evoked potentials (16). Therefore, patients with both depression and sleep disturbances may have further impaired plasticity processes that hinder rTMS effects. Following this logic, improving sleep through medications, such as hypnotics, may help restore plasticity mechanisms and aid rTMS treatment.

One promising biomarker that may reveal greater insight into the relationship between sleep, depression, and rTMS is brain-derived neurotrophic factor (BDNF). The role of BDNF in synaptic plasticity has been well-established (39) and BDNF levels have been shown to increase alongside slow-wave activity (SWA) following ketamine therapy for depression specifically in responders (40). BDNF polymorphism has also demonstrated a potential influence on rTMS-induced memory performance (41). Furthermore, work in rodents show increased SWA following BDNF injection that can be blocked by administration of BDNF receptor inhibitors (42). Taken together, BDNF levels may offer a physiological signal to quantify both sleep quality in correlation with SWA and underlying plasticity modulating TMS treatment outcome.

Several meta-analyses have reported that concurrent cognitive-behavioral therapy for insomnia (CBT-I) may improve the efficacy of anti-depressant treatment (26, 43, 44). No studies have yet examined targeted CBT-I treatment alongside rTMS, with the exception of one open-label feasibility trial with 2 patients undergoing a 36-day TMS treatment course (10-Hz, 3,000 pulses) with six weekly 1-hour manualized CBT-I sessions (45). Both patients experienced significant improvement in subjective sleep rating and reached remission as measured by the 24-item Hamilton Rating Scale for Depression-24 (HRSD_24_). Our findings provide support that treating sleep alongside TMS treatment may produce synergistic antidepressant effects. Prospective work is needed to determine if improving sleep improves TMS outcomes, both with medications and non-pharmaceutical interventions such as cognitive-behavior therapy for insomnia.

Our interpretations are limited by confounding variables that cannot be controlled with retrospective, naturalistic data, such as the wide variety of medication regimes that have varying effects on sleep. Lifestyle factors have also been found to influence TMS-induced plasticity and potentially TMS outcomes. In preclinical studies, individuals with chronic caffeine use have demonstrated blunted motor plasticity following rTMS relative to non-caffeine users (46), while musicians and athletes exhibit enhanced rTMS-induced motor plasticity (47). We also did not include in our filter criteria other drugs that are not strictly categorized as sleep medications but commonly used to treat sleep, such as benzodiazepines or marijuana. As a result, it is difficult to definitively claim that treating sleep with medications improves TMS outcome. Sleep medications also impact motor threshold and may impact the intensity of stimulation delivered for TMS treatment. Finally, we did not have data regarding length, timing, or dosages of sleep medication use, which limits our ability to conclusively determine a causal relationship with and TMS outcomes.

A percentage of the sample received predominantly 5-Hz rTMS, or iTBS, which also may have differential effects on sleep and depression. rTMS delivered at 5-Hz and greater is considered “excitatory” and considered identical in clinical application. However, research has demonstrated that parameters such as frequency, pulse number or train duration can influence or even reverse effects on brain excitability (48). Therefore, we cannot state that these various forms of excitatory rTMS work through identical pathways to improve depression and sleep. An exploration of the potential relationship between TMS parameters and sleep (and depression) is still needed.

A major limitation of our study is that there were no objective measures of sleep. Past studies have previously reported discrepancies between subjective and objective ratings of sleep quality. Future studies would benefit from including objective measures such as actigraphy and EEG-based quality and quantity of sleep architecture or changes in brain states to corroborate subjective sleep experience and perhaps yield new insight into how treatment of sleep during TMS may predict overall depression improvement to treatment. The creation of the dichotomous “no/low” and “high” insomnia groups using the mean insomnia score of 4, in line with the method used in Fava et al. (2002), may not be an accurate categorization of insomnia severity (49). An individual who scored a 3 on a single item would be categorized by our methods as “no/low” insomnia, though endorsing for example, “I take more than 60 minutes to fall asleep, more than half the time.” As insomnia involves different dimensions of sleep, and there are interindividual differences in phenotype, binary categorization of severity presents a challenge.

Understanding what factors influence TMS treatment and in what direction are critical for patients to maximize the benefits of TMS. Our findings suggest that while baseline sleep quality is not predictive of TMS clinical outcomes, modulating sleep may impact the trajectory of symptom improvement during a treatment course.

## Data Availability

Data set includes identifiable information and is overseen by the Butler Hospital TMS Clinic. Requests to access these datasets should be directed to AGobin@butler.org.

## Supplementary material

Supplementary Figure 1 Histogram of baseline insomnia score.
