# Supplementary figures and images for: Effect of sleep quality on repetitive transcranial magnetic stimulation outcomes in depression
Source: Front Psychiatry. 2024 Sep 23;15:1458696. doi: 10.3389/fpsyt.2024.1458696 (PMC11456523; doi:10.3389/fpsyt.2024.1458696)

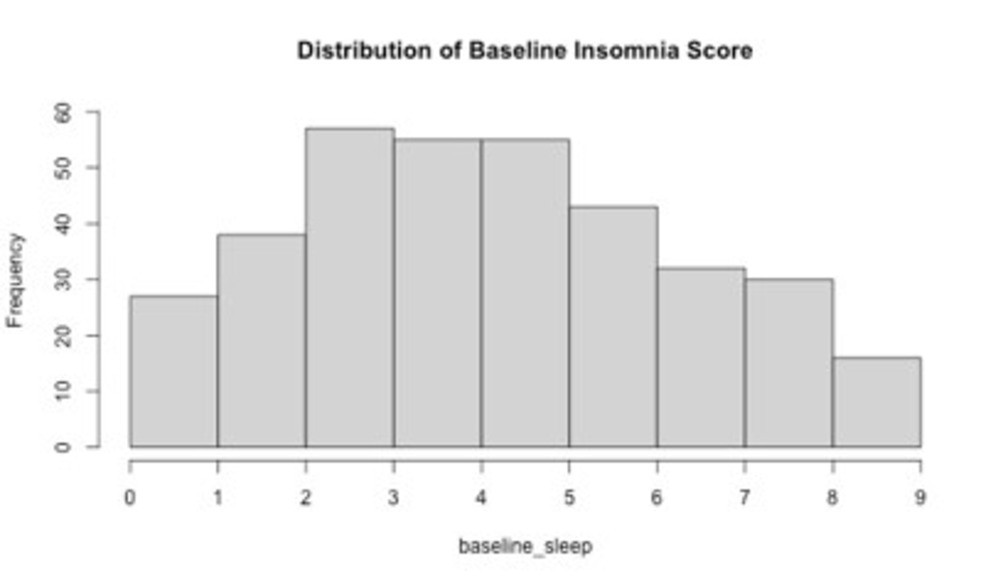

Supplement: Supplementary Figure 1 — Histogram of baseline insomnia score. [file Image1.jpeg]
